# Type III Secretion System Genotypes and Biofilm-Forming Capacity of *Pseudomonas aeruginosa* Bloodstream Isolates: An Exploratory Analysis of the *exoU*–Fluoroquinolone Association

**DOI:** 10.3390/pathogens15101052

**Published:** 2026-10-04

**Authors:** Hazel Malaş İren, Ergun Mete

**Affiliations:** 1 Department of Medical Microbiology, Denizli State Hospital, Merkezefendi, 20010 Denizli, Turkey; hazel-20-07@hotmail.com; 2 Department of Medical Microbiology, Faculty of Medicine, Pamukkale University, Pamukkale, 20160 Denizli, Turkey

**Keywords:** *Pseudomonas aeruginosa*, type III secretion system, *exoU*, biofilm, bloodstream infection, fluoroquinolone resistance

## Abstract

The pathogenicity of *Pseudomonas aeruginosa* in bloodstream infection is shaped by biofilm formation and the type III secretion system (T3SS). We characterised the biofilm-associated and T3SS genotypes of 100 non-duplicate bloodstream isolates and tested whether either trait co-segregates with antimicrobial resistance. Biofilm was assessed by Congo red agar (CRA) and the quantitative microtiter plate (MTP) assay; biofilm-associated (*pslA*, *pelA*, *ppyR*) and T3SS effector (*exoS*, *exoY*, *exoT*, *exoU*) genes by conventional PCR; and susceptibility by an automated system interpreted with EUCAST criteria. Biofilm was detected in 84% of isolates by MTP and 25% by CRA (Cohen’s κ = 0.16). *pslA* was present in 100%, *pelA* and *ppyR* in 99%; all isolates carried *exoY* and *exoT*, *exoS* was found in 88% and *exoU* in 27%. Multidrug resistance was 15%. Biofilm phenotype was unrelated to resistance. In contrast, levofloxacin resistance was more frequent among *exoU*-positive isolates (29.6% vs. 9.6%; *p* = 0.024), and this association survived adjustment for biofilm phenotype and hospital unit in multivariable analysis (adjusted OR 3.90, 95% CI 1.20–12.67). However, the association did not remain significant after correction for multiple comparisons or after excluding *exoS*/*exoU* co-positive isolates. The *exoU*–fluoroquinolone relationship is therefore presented as an exploratory, hypothesis-generating finding that requires confirmation by reference susceptibility testing and clonal characterisation.

## 1. Introduction

*Pseudomonas aeruginosa* is a Gram-negative, non-fermenting opportunistic pathogen and a leading cause of hospital-acquired infection worldwide. Its clinical importance derives from a large accessory genome [1], rapid acquisition of antimicrobial resistance [2], and an extensive arsenal of virulence determinants that together permit survival in hostile environments and evasion of both innate and adaptive immunity [3,4,5,6]. Bloodstream infection with this organism carries high mortality [7], and the determinants distinguishing more from less virulent bloodstream isolates remain incompletely defined [5].

Two virulence traits are particularly relevant. The first is biofilm formation: within a self-produced matrix of exopolysaccharides, extracellular DNA and proteins, biofilm-embedded cells grow slowly, resist phagocytosis and are shielded from antimicrobial penetration; the Psl and Pel polysaccharides, encoded by the *psl* and *pel* operons, are central to this architecture, and *ppyR* contributes to their regulation [8,9,10]. The second is the T3SS, a needle-like apparatus injecting effector proteins into host cells. Four effectors occur in *P. aeruginosa*: ExoS, ExoT, ExoY and ExoU [4]. ExoU, a potent phospholipase, is associated with acute cytotoxicity and worse outcome, and *exoU* and *exoS* are usually described as mutually exclusive, defining two major lineages.

Whether these traits co-segregate with antimicrobial resistance is clinically important, because it would allow one property to serve as a partial marker of the other. Reports linking biofilm capacity to resistance are inconsistent, whereas an association between the *exoU* genotype and fluoroquinolone resistance has been described in several settings [11]. We therefore characterised the biofilm-associated and T3SS genotypes of 100 *P. aeruginosa* bloodstream isolates. Specifically, we detected the biofilm-associated genes *pslA*, *pelA* and *ppyR* and the T3SS effector genes *exoS*, *exoY*, *exoT* and *exoU* by conventional PCR, quantified biofilm formation by two independent assays (Congo red agar and the microtiter plate method), and determined antimicrobial susceptibility interpreted by EUCAST criteria. We then tested whether biofilm phenotype or T3SS genotype was associated with resistance to commonly used antipseudomonal agents, and examined the *exoU*–fluoroquinolone relationship in a multivariable model.

## 2. Materials and Methods

### 2.1. Study Design, Setting and Ethics

This was a cross-sectional, descriptive study on stored clinical isolates collected prospectively during routine diagnostic activity between 2018 and 2019 in the clinical microbiology laboratory of a tertiary-care university hospital and archived at −80 °C. The molecular and phenotypic characterisation reported here was approved by the Non-Interventional Clinical Research Ethics Committee of Pamukkale University (25 December 2019, decision no. E.92290). Patient identifiers were removed before analysis; the requirement for individual consent was waived because the study used archived isolates without identifiable patient data.

### 2.2. Bacterial Isolates

One hundred consecutive, non-duplicate *P. aeruginosa* blood-culture isolates were included. By clinical unit of origin, 53 were from intensive-care units (including the coronary intensive-care unit) and 47 from general inpatient wards. Only the first isolate per patient was retained. Isolates were identified by colony morphology, Gram stain and oxidase reaction, confirmed with the BD Phoenix system (Becton Dickinson, Sparks, MD, USA); *P. aeruginosa* PAO1 served as reference strain.

### 2.3. Antimicrobial Susceptibility Testing

Susceptibility was determined with the BD Phoenix automated system. This instrument does not perform reference broth microdilution: growth across a limited concentration series is monitored turbidimetrically and colorimetrically, and an estimated MIC and category are derived algorithmically. The values reported here are therefore instrument-derived estimates, not reference MICs. The results were interpreted with EUCAST breakpoint tables v9.0 (2019) [12]. The agents evaluated were cefepime, ceftazidime, piperacillin–tazobactam, aztreonam, imipenem, meropenem, ciprofloxacin, levofloxacin, gentamicin, amikacin and netilmicin. Multidrug resistance was defined as non-susceptibility to ≥1 agent in ≥3 antipseudomonal classes.

### 2.4. Biofilm Detection by Congo Red Agar

CRA was prepared from a brain–heart infusion base with sucrose and agar; Congo red was dissolved and autoclaved separately and added to the cooled medium. A single colony from a pure blood-agar subculture was streaked onto CRA and incubated aerobically at 37 °C for 24 h. Dry, crystalline, black colonies were scored positive and smooth lighter colonies negative. PAO1 was included on every batch. On CRA, Congo red binds the secreted exopolysaccharide/slime matrix, so a dry, crystalline, black colony is the established phenotypic indicator of slime (biofilm-matrix) production, whereas non-producers form smooth pink-to-red colonies; this interpretation follows the criteria originally described by Freeman et al. [13] and applied in later biofilm studies [14].

### 2.5. Quantitative Biofilm Assay (Microtiter Plate)

Biofilm biomass was quantified in 96-well polystyrene plates. Overnight cultures were adjusted to 0.5 McFarland and dispensed into wells, with sterile broth as the negative control and PAO1 as the reference [15,16]. After 24 h at 37 °C, the wells were aspirated, washed with PBS, air-dried, stained with 0.1% crystal violet, washed, solubilised in acetic acid and read at 590 nm [16,17,18]. Each isolate was tested in three independent experiments. The cut-off OD (ODc) was the mean of negative controls plus three standard deviations; isolates were categorised as non-producer (OD ≤ ODc), weak (ODc < OD ≤ 2 × ODc), moderate (2 × ODc < OD ≤ 4 × ODc) or strong (OD > 4 × ODc) [16].

For comparative analyses, strong and moderate producers were grouped as biofilm-positive (*n* = 69) and weak and non-producers as biofilm-negative (*n* = 31). This clinically oriented dichotomy was preferred over the simple OD > ODc threshold (which classifies 84 isolates as positive) because weak adherence cannot be reliably distinguished from background in a single-species assay.

### 2.6. DNA Extraction and PCR

Genomic DNA was extracted by boiling: a single colony was grown in broth at 37 °C for 18–24 h, the pellet resuspended in sterile water and heat-lysed, and the supernatant after high-speed centrifugation used as a template, stored at −20 °C [19]. Template adequacy was confirmed by amplification of *pslA* in all isolates. Biofilm-associated (*pslA*, *pelA*, *ppyR*) and T3SS effector (*exoS*, *exoY*, *exoT*, *exoU*) genes were amplified by conventional PCR with published primers (Table 1) in 25 µL reactions (buffer, dNTPs, MgCl_2_, Taq polymerase, primers, template). Cycling: 94 °C 5 min; 30–35 cycles of 94 °C 30 s, 55 °C 30 s, 72 °C 1 min; final 72 °C 5 min. Products were resolved on 1% agarose with ethidium bromide, sized against a 100 bp ladder and imaged (Gel Logic 2200, Kodak, Rochester, NY, USA). *P. aeruginosa* PAO1 (*exoS*-positive, *exoU*-negative) was used as the reference strain and a no-template reaction as the negative control [20,21]. A dedicated *exoU*-positive control strain (e.g., PA14) was not available; *exoU* positivity was therefore scored solely on the presence of an amplicon of the expected size, a limitation addressed in Section 4.4.

### 2.7. Statistical Analysis

Analyses used SPSS v25 (IBM, Armonk, NY, USA) and were verified in Python 3.11 (SciPy 1.17.1, statsmodels 0.15.0). Categorical variables are counts and percentages; groups were compared by Fisher’s exact test. Agreement between CRA and MTP was assessed by the McNemar test and quantified by Cohen’s κ. The independence of the *exoU*–levofloxacin association was examined by multivariable logistic regression with biofilm phenotype and hospital unit as covariates. A two-sided *p* < 0.05 was significant. Because resistance to eight agents was tested against three grouping variables, the genotype–resistance comparisons are exploratory. For the *exoU* subgroup, the eight *exoU*-positive versus *exoU*-negative antibiotic comparisons were corrected for multiple testing using the Benjamini–Hochberg false discovery rate; raw and FDR-corrected *p*-values are reported in Table S3.

## 3. Results

### 3.1. Biofilm Production and Distribution by Hospital Unit

By MTP, 84/100 isolates (84%) produced biofilm above the ODc threshold (24 strong, 45 moderate, 15 weak); 16 were non-producers. Under the strong-or-moderate definition, 69 isolates (69%) were biofilm-positive. By CRA, only 25 (25%) were positive. CRA positivity was significantly more frequent among intensive-care isolates (19/53, 35.8%) than ward isolates (6/47, 12.8%; *p* = 0.011); this difference was not reproduced by the quantitative assay, in which biofilm production was comparable between settings (Table 2; *p* = 0.832).

### 3.2. Agreement Between the Two Biofilm Assays

Using the strong-or-moderate definition, 47 isolates were biofilm-positive by MTP but negative by CRA, whereas only three showed the reverse pattern (concordant: 22 double-positive, 28 double-negative). Agreement beyond chance was poor (Cohen’s κ = 0.16), and the McNemar test confirmed a strong systematic discrepancy (*p* < 0.001): CRA substantially under-detects biofilm production in this collection rather than disagreeing at random.

### 3.3. Biofilm-Associated and T3SS Genotypes

**Figure 1:**
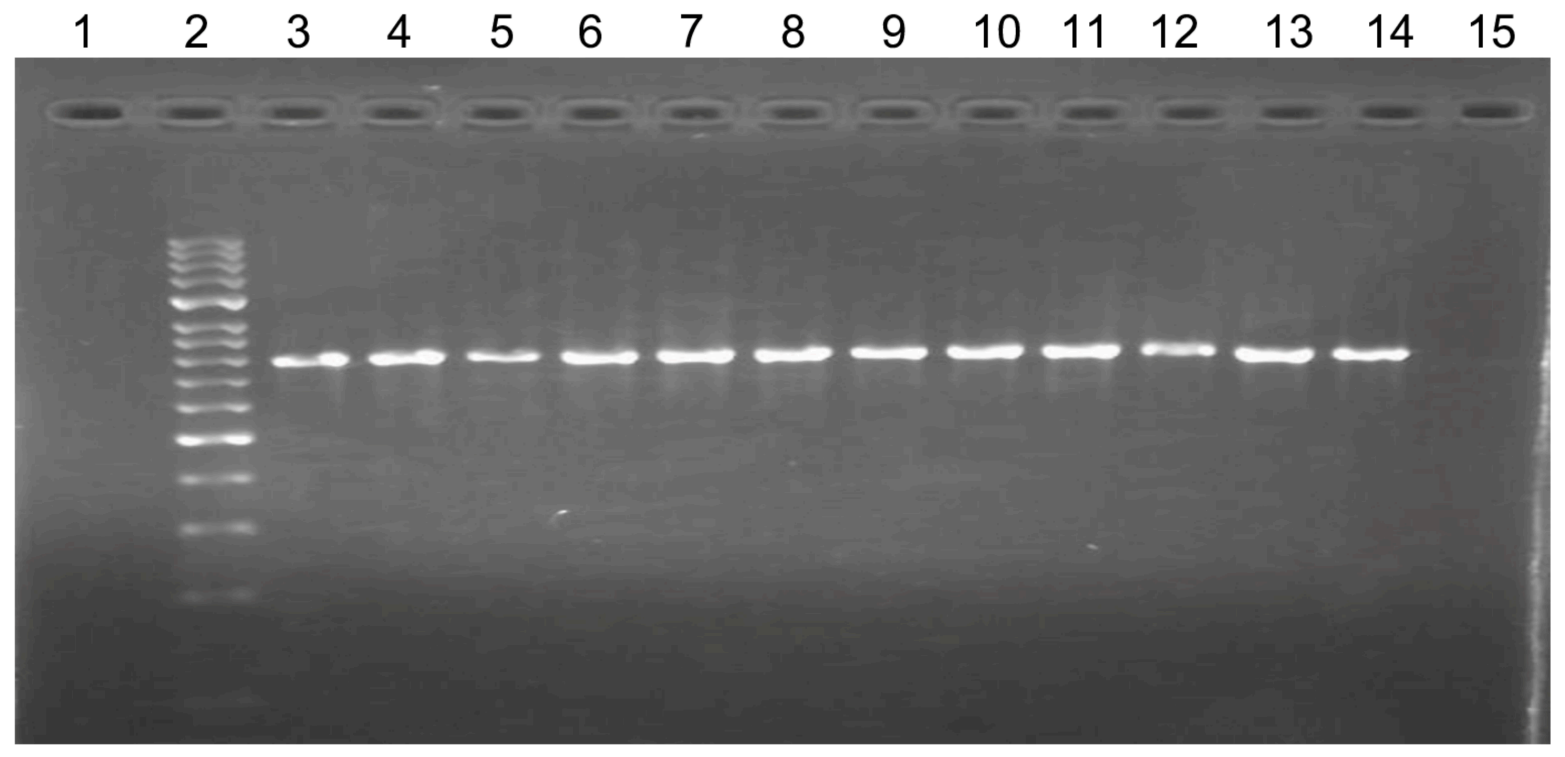
Representative agarose gel electrophoresis of the *pelA* gene (786 bp). Lane 1: the negative control; lane 2: the 100 bp marker; lane 3: the positive control strain PAO1; lanes 4–14: the *pelA*-positive clinical isolates; lane 15: the *pelA*-negative clinical isolate. Each isolate was tested once by conventional end-point PCR; the gel shown is representative of all 100 isolates.

**Figure 2:**
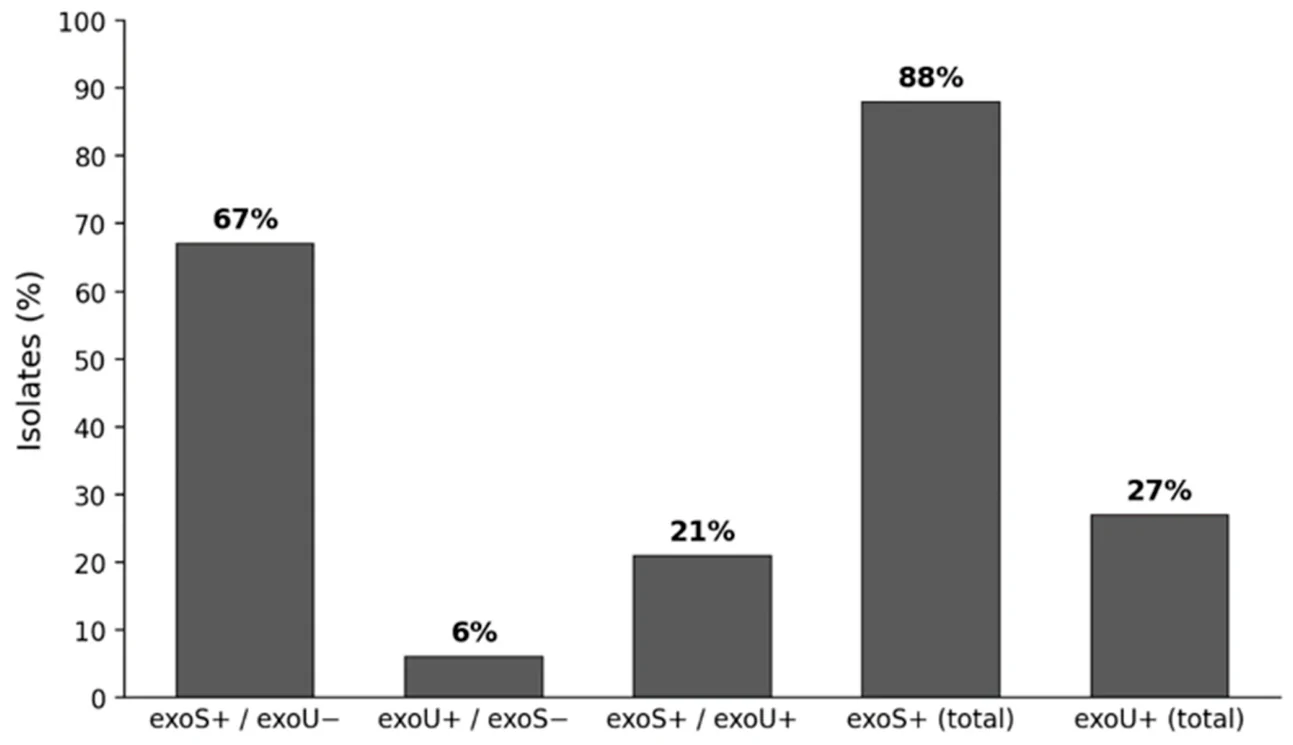
The distribution of *exoS* and *exoU* genotypes among the 100 *P. aeruginosa* bloodstream isolates. *exoY* and *exoT* were present in all isolates and are not shown. The bars show the percentage of the 100 isolates with each genotype, based on single end-point PCR detection per isolate; the figure is descriptive and no statistical test was applied to these proportions.

*pslA* was detected in all 100 isolates, *pelA* and *ppyR* in 99 each; 99 isolates carried all three. A representative *pelA* PCR gel is shown in Figure 1. All isolates were positive for *exoY* and *exoT* (100% each), so neither could serve as a grouping variable and both were excluded from Table 3. *exoS* was present in 88 isolates and *exoU* in 27. The *exoS*/*exoU* cross-tabulation gave: *exoS*+/*exoU*− = 67, *exoS*+/*exoU*+ = 21, *exoS*−/*exoU*+ = 6 and *exoS*−/*exoU*− = 6 (Figure 2).

### 3.4. Antimicrobial Susceptibility

Resistance rates were: ceftazidime 20%, imipenem 19%, piperacillin–tazobactam 18%, cefepime 17%, levofloxacin 15%, ciprofloxacin 12%, meropenem 12%, gentamicin 6%, aztreonam 2%, netilmicin 2% and amikacin 1% (Table S1). Fifteen isolates (15%) were multidrug-resistant. The very low aminoglycoside (amikacin, netilmicin) and aztreonam resistance indicates these agents retained good in vitro activity against the collection; because so few isolates were resistant, these three agents were not included in the subgroup analyses of Table 3.

### 3.5. Biofilm Phenotype, T3SS Genotype and Resistance

Biofilm phenotype was not significantly associated with resistance to any agent; although several β-lactams showed numerically higher resistance among biofilm-positive isolates, no comparison approached significance (Table 3). In contrast, the T3SS genotype discriminated fluoroquinolone susceptibility. Levofloxacin resistance was significantly more frequent in *exoU*-positive than *exoU*-negative isolates (8/27, 29.6% vs. 7/73, 9.6%; *p* = 0.024), with the same direction for ciprofloxacin (6/27, 22.2% vs. 6/73, 8.2%; *p* = 0.081). A reciprocal nominal association was seen between *exoS*-negativity and ciprofloxacin resistance (4/12, 33.3% vs. 8/88, 9.1%; *p* = 0.036). This subgroup was very small: only 12 isolates were *exoS*-negative, of which six (50%) were also *exoU*-positive. The *exoS*-negativity signal therefore overlaps only in part with the *exoU* genotype and cannot be cleanly separated from it; given the small numbers, we treat this reciprocal association as exploratory. Here, “overlap” refers to co-classification in the data, not to a molecular interaction between the genes.

In multivariable logistic regression with levofloxacin resistance as the outcome, the *exoU* genotype remained associated with levofloxacin resistance after adjustment for biofilm phenotype and hospital unit (an exploratory adjusted association, not a confirmed independent predictor) (adjusted odds ratio [OR] 3.90, 95% confidence interval [CI] 1.20–12.67; *p* = 0.024); neither biofilm phenotype (OR 0.41, 95% CI 0.12–1.34) nor intensive-care origin (OR 2.53, 95% CI 0.71–8.97) was significant. The full model is given in Table S2. The association persisted after adjustment for these measured covariates; however, residual confounding cannot be excluded. The wide confidence interval reflects the small number of resistant events (15 events across three covariates), so the model is best interpreted as exploratory. To assess whether the *exoU*–levofloxacin association depended on the *exoS*/*exoU* co-positive subset, a sensitivity analysis was performed after excluding the 21 co-positive isolates. Among the remaining 79 isolates, levofloxacin resistance was still more frequent in *exoU*-positive than *exoU*-negative isolates (2/6, 33.3% vs. 7/73, 9.6%), preserving the direction and magnitude of the effect; however, with only six *exoU*-positive isolates remaining, the comparison was underpowered and did not reach significance (Fisher *p* = 0.136) (Table S4). In addition, when the eight antibiotic comparisons in the *exoU* subgroup were corrected for multiple testing (Benjamini–Hochberg false discovery rate), no comparison remained significant, including levofloxacin (FDR-adjusted *p* = 0.19; Table S3). These analyses indicate that the *exoU*–levofloxacin signal is consistent in direction but does not survive correction for multiplicity or subgroup restriction, and should therefore be regarded as exploratory and hypothesis-generating.

## 4. Discussion

The central observation is a dissociation: among bloodstream *P. aeruginosa*, biofilm-forming capacity did not predict antimicrobial resistance, whereas the *exoU* genotype was associated with levofloxacin resistance in exploratory adjusted analysis. The two virulence traits therefore behave differently as epidemiological markers, arguing against treating “virulence” as a single axis parallel to resistance.

### 4.1. exoU and Fluoroquinolone Resistance

*exoU*-positive isolates were about three times more likely to be levofloxacin-resistant, and the association survived adjustment for biofilm phenotype and hospital unit (adjusted OR 3.90). Its magnitude and direction match Wong-Beringer et al., who reported higher levofloxacin resistance among *exoU*-positive than *exoS*-positive isolates, with *gyrA* mutations in 91% of *exoU*-positive isolates and an efflux-overexpressing phenotype [11]. Two non-exclusive explanations exist. The first is clonal: *exoU* lies on a genomic island carried by particular high-risk clones that have independently accumulated fluoroquinolone-resistance determinants, so the association would mark lineage. The second is mechanistic: fluoroquinolone-induced *gyrA* mutations alter DNA supercoiling, which modulates T3SS gene expression [11]. Our data cannot separate these, because clonal relatedness was not determined; this is the principal remaining gap. If confirmed, this pattern would carry a clinical implication: empirical fluoroquinolone therapy for *P. aeruginosa* bacteraemia might be least effective against the isolates of greatest cytotoxic potential; however, given the exploratory nature of this finding, this possibility requires confirmation with reference susceptibility testing and linked clinical outcomes before it can inform empirical therapy. Consistent with this, *exoU*-positive strains have been reported to carry quinolone resistance–determining region (QRDR) mutations in *gyrA* (e.g., Thr83Ile) and *parC* (e.g., Ser87Leu) more frequently than *exoS*-positive strains [22], and such topoisomerase QRDR mutations are a principal determinant of fluoroquinolone resistance in *P. aeruginosa* [23].

### 4.2. Distribution of T3SS Genotypes and the exoS/exoU Co-Positive Subset

Universal *exoT* and *exoY* carriage is consistent with their core-genome location and with most surveys [24,25]. The *exoU* frequency of 27% falls within the usual clinical range of roughly 20–40%. Our most striking genotypic observation was the co-detection of *exoS* and *exoU* in 21% of isolates. These two effectors are classically regarded as almost mutually exclusive, defining the invasive and cytotoxic lineages respectively [11,26], and in several clinical collections co-carriage is uncommon; a comparable 100-isolate series from our own region reported only 2% [24]. A rate as high as ours therefore calls for careful interpretation.

Two lines of evidence indicate that a genuine *exoS*+/*exoU*+ subset exists and is clinically relevant, rather than being merely an artefact. First, in a series of 189 clinical isolates from Peruvian hospitals, *exoS*, *exoT* and *exoY* were present in all isolates while *exoU* occurred in 22.8%, necessarily producing a high *exoS*+/*exoU*+ frequency; this genotype was distributed across eight sequence types and was associated with multidrug resistance and higher-level quinolone resistance [27]. Second, recent functional work has characterised *exoS*+/*exoU*+ strains as an emerging hypervirulent subset that co-expresses and co-secretes both effectors, with *exoU* acquired by horizontal transfer of the PAPI-2 pathogenicity island onto an *exoS*+ background, and such strains have been reported worldwide, including within high-risk clones such as ST235 [28]. Our 21% co-positive rate is compatible with this emerging picture and, if confirmed, would be a clinically meaningful observation for bloodstream isolates given the association of this genotype with resistance and severe infection.

Nonetheless, this interpretation must remain provisional. We detected gene presence by PCR only and did not perform molecular typing (MLST) or sequencing, so we cannot exclude a contribution from non-specific amplification or confirm the clonal context of the co-positive isolates. Sequencing or MLST of these 21 isolates is the necessary next step and is acknowledged as a limitation (Section 4.4).

### 4.3. Biofilm: Assay Discordance and Absence of a Resistance Association

The two biofilm assays agreed poorly (κ = 0.16), with 47 isolates positive by MTP alone. This fits reports that CRA has high specificity but low sensitivity relative to MTP, the more reliable quantitative approach [16,29], and supports restricting CRA to crude screening. The higher CRA positivity among intensive-care isolates (35.8% vs. 12.8%) was not reproduced by the quantitative assay, so it is better read as an artefact of the less sensitive method than as a real difference; had we relied on CRA alone, we would have reached a misleading conclusion. Despite near-universal *pslA*, *pelA* and *ppyR*, biofilm phenotype varied widely, which confirms that gene carriage is a poor proxy for expressed phenotype. The same caveat applies to the T3SS genes, since detection indicates genetic potential and not secretion of functional protein. No association was found between biofilm phenotype and resistance to any agent, in line with the divided reports in the literature [4,30,31]. Biofilm-associated tolerance is a property of the sessile state, arising from delayed matrix penetration, slow growth and an altered microenvironment [32], and is not expected to surface when planktonic cells are tested; with only 31 biofilm-negative isolates, the study also had limited power to detect modest differences.

### 4.4. Limitations

This study has several limitations. Susceptibility was determined with an automated system that estimates MICs algorithmically rather than by reference broth microdilution or disc diffusion; EUCAST criteria were applied, but the categorical results were not confirmed by a reference method, and this is the principal methodological constraint. We did not investigate clonal relatedness, so we cannot say whether the *exoU*–fluoroquinolone association reflects the spread of one or a few clones. Gene detection relied on conventional PCR without a dedicated *exoU*-positive control strain, since only PAO1 (which is *exoU*-negative) was available, and without sequence confirmation, so *exoU* positivity was scored on amplicon size alone. This last point bears directly on the unexpectedly high *exoS*+/*exoU*+ co-detection rate discussed in Section 4.2, which may in part reflect non-specific amplification and should be verified by sequencing or MLST. We assessed gene presence but not expression, and we could not link clinical outcomes to individual isolates, which precludes any analysis of *exoU* against disease severity or acute-versus-chronic patterns. The isolates date from 2018 to 2019 and predate recent shifts in resistance epidemiology, although genotype distributions are likely more stable over time than resistance rates. Finally, the logistic model rests on only 15 resistant isolates, which explains the wide confidence interval, and the Table 3 comparisons are reported as raw *p*-values, with FDR-corrected values for the *exoU* comparisons in Table S3, so the nominally significant results should be treated as hypothesis-generating.

## 5. Conclusions

In 100 *P. aeruginosa* bloodstream isolates, biofilm-associated genes were nearly universal while expressed biofilm capacity varied widely, and biofilm phenotype was unrelated to resistance. The *exoU* genotype, present in just over a quarter of isolates, showed a consistent association with higher levofloxacin resistance. In exploratory analyses this signal was carried by genotype rather than biofilm phenotype; however, it did not survive correction for multiple comparisons or exclusion of the *exoS*/*exoU* co-positive isolates. We therefore present this as a hypothesis-generating observation that requires confirmation by reference susceptibility testing and clonal (MLST or sequencing) characterisation in a collection with linked clinical outcomes.

## Figures and Tables

**Table 1:** Primers used for amplification of biofilm-associated and T3SS genes.

Gene	Primer Sequence (5′ → 3′)	Amplicon (bp)	Ref.
*pslA*	F: TCCCTACCTCAGCAGCAAGC/R: TGTTGTAGCCGTAGCGTTTCTG	656	[21]
*pelA*	F: CATACCTTCAGCCATCCGTTCTTC/R: CGCATTGCCGCACTCAG	786	[21]
*ppyR*	F: CGTGATCGCCGCCTATTTCC/R: ACAGCAGACCTCCCAACCG	160	[21]
*exoS*	F: GCGAGGTCAGCAGAGTATCG/R: TTCGGCGTCACTGTGGATGC	118	[20]
*exoY*	F: CGGATTCTATGGCAGGGAGG/R: GCCCTTGATGCACTCGACCA	289	[20]
*exoT*	F: AATCGCCGTCCAACTGCATGCG/R: TGTTCGCCGAGGTACTGCTC	152	[20]
*exoU*	F: CCGTTGTGGTGCCGTTGAAG/R: CCAGATGTTCACCGACTCGC	134	[20]

**Table 2:** Biofilm production of the 100 bloodstream isolates by hospital unit.

Biofilm Category	ICU (*n* = 53)	Ward (*n* = 47)	Total (*n* = 100)	*p*
Microtiter plate assay				
Strong	10 (18.9)	14 (29.8)	24 (24.0)	
Moderate	26 (49.1)	19 (40.4)	45 (45.0)	
Weak	7 (13.2)	8 (17.0)	15 (15.0)	
Non-producer	10 (18.9)	6 (12.8)	16 (16.0)	
Biofilm-positive (strong + moderate)	36 (67.9)	33 (70.2)	69 (69.0)	0.832
Congo red agar assay				
Positive	19 (35.8)	6 (12.8)	25 (25.0)	0.011
Negative	34 (64.2)	41 (87.2)	75 (75.0)	

Data are *n* (%). *p* by Fisher’s exact test.

**Table 3:** Antimicrobial resistance by biofilm phenotype and T3SS genotype (Fisher’s exact test).

Antimicrobial	Biofilm Phenotype			*exoU* Genotype			*exoS* Genotype		
	Pos (*n* = 69)	Neg (*n* = 31)	*p*	Pos (*n* = 27)	Neg (*n* = 73)	*p*	Pos (*n* = 88)	Neg (*n* = 12)	*p*
Cefepime	14 (20.3)	3 (9.7)	0.256	7 (25.9)	10 (13.7)	0.228	15 (17.0)	2 (16.7)	1.000
Ceftazidime	17 (24.6)	3 (9.7)	0.108	7 (25.9)	13 (17.8)	0.404	16 (18.2)	4 (33.3)	0.251
Piperacillin–tazobactam	14 (20.3)	4 (12.9)	0.574	8 (29.6)	10 (13.7)	0.082	15 (17.0)	3 (25.0)	0.448
Imipenem	15 (21.7)	4 (12.9)	0.411	6 (22.2)	13 (17.8)	0.774	18 (20.5)	1 (8.3)	0.454
Meropenem	8 (11.6)	4 (12.9)	1.000	5 (18.5)	7 (9.6)	0.297	11 (12.5)	1 (8.3)	1.000
Ciprofloxacin	7 (10.1)	5 (16.1)	0.507	6 (22.2)	6 (8.2)	0.081	8 (9.1)	4 (33.3)	0.036 *
Levofloxacin	8 (11.6)	7 (22.6)	0.224	8 (29.6)	7 (9.6)	0.024 *	13 (14.8)	2 (16.7)	1.000
Gentamicin	3 (4.3)	3 (9.7)	0.371	3 (11.1)	3 (4.1)	0.339	5 (5.7)	1 (8.3)	0.545

The data are the number (%) of resistant isolates. Biofilm-positive = strong or moderate producer by MTP. *exoY* and *exoT* omitted (both present in all 100 isolates). * *p* < 0.05 (raw *p*-values; FDR-corrected *p*-values for the *exoU* comparisons are given in Table S3).

## Data Availability

The data supporting the findings of this study are available from the corresponding author upon reasonable request.

## Supplementary Materials

The following supporting information can be downloaded at: https://www.mdpi.com/article/10.3390/pathogens15101052/s1. Table S1: Antimicrobial resistance rates of the 100 *Pseudomonas aeruginosa* bloodstream isolates, interpreted by EUCAST breakpoint tables v9.0 (2019); Table S2: Multivariable logistic regression model for levofloxacin resistance (*n* = 100; 15 resistant events); Table S3: Association between *exoU* genotype and antimicrobial resistance (*exoU*-positive vs. *exoU*-negative), with Benjamini–Hochberg correction for multiple comparisons; Table S4: Sensitivity analysis: *exoU* genotype and levofloxacin resistance after excluding the 21 *exoS*/*exoU* co-positive isolates.
