# Supplementary material for: Type III Secretion System Genotypes and Biofilm-Forming Capacity of Pseudomonas aeruginosa Bloodstream Isolates: An Exploratory Analysis of the exoU–Fluoroquinolone Association
Source: Pathogens. 2026 Oct 4;15(10):1052. doi: 10.3390/pathogens15101052 (PMC13708411; doi:10.3390/pathogens15101052)
Supplement: Supplementary file 1 [file pathogens-15-01052-s001.zip › pathogens-4546490-supplementary.pdf]

Supplementary Materials

Type III Secretion System Genotypes and Biofilm-Forming Capacity of Pseudomonas aeruginosa Bloodstream Isolates: An Exploratory Analysis of the exoU–Fluoroquinolone Association

Table S1. Antimicrobial resistance rates of the 100 *Pseudomonas aeruginosa* bloodstream isolates, interpreted by EUCAST breakpoint tables v9.0 (2019).

Antimicrobial agent	Class	Resistant, n (%)
Cefepime	Cephalosporin	17 (17)
Ceftazidime	Cephalosporin	20 (20)
Piperacillin–tazobactam	β-lactam/inhibitor	18 (18)
Aztreonam	Monobactam	2 (2)
Imipenem	Carbapenem	19 (19)
Meropenem	Carbapenem	12 (12)
Ciprofloxacin	Fluoroquinolone	12 (12)
Levofloxacin	Fluoroquinolone	15 (15)
Gentamicin	Aminoglycoside	6 (6)
Amikacin	Aminoglycoside	1 (1)
Netilmicin	Aminoglycoside	2 (2)

Multidrug resistance (non-susceptibility to ≥1 agent in ≥3 antipseudomonal classes) was present in 15 isolates (15%).

Table S2. Multivariable logistic regression model for levofloxacin resistance (n = 100; 15 resistant events).

Variable	Adjusted OR	95% CI	p
exoU genotype (positive)	3.90	1.20–12.67	0.024
Biofilm phenotype (positive)	0.41	0.12–1.34	0.139
Intensive-care origin	2.53	0.71–8.97	0.151

OR, odds ratio; CI, confidence interval. Outcome: levofloxacin resistance. The model rests on 15 resistant events; the wide confidence interval reflects this limited number.

Table S3. Association between *exoU* genotype and antimicrobial resistance (*exoU*-positive vs. *exoU*-negative), with Benjamini–Hochberg correction for multiple comparisons.

Antimicrobial agent	*exoU*+ vs *exoU*– (%)	Raw p	FDR p
Cefepime	25.9 vs 13.7	0.228	0.452
Ceftazidime	25.9 vs 17.8	0.404	0.462
Piperacillin–tazobactam	29.6 vs 13.7	0.082	0.218
Imipenem	22.2 vs 17.8	0.774	0.774
Meropenem	18.5 vs 9.6	0.297	0.452
Ciprofloxacin	22.2 vs 8.2	0.081	0.218
Levofloxacin	29.6 vs 9.6	0.024	0.190
Gentamicin	11.1 vs 4.1	0.339	0.452

FDR, false discovery rate (Benjamini–Hochberg). After correction for the eight comparisons, no association remained significant, including levofloxacin (FDR p = 0.19).

Table S4. Sensitivity analysis: *exoU* genotype and levofloxacin resistance after excluding the 21 *exoS*/*exoU* co-positive isolates.

Analysis	*exoU*+ vs *exoU*– (%)	Fisher p
All isolates (n = 100)	29.6 (8/27) vs 9.6 (7/73)	0.024
Excluding co-positive (n = 79)	33.3 (2/6) vs 9.6 (7/73)	0.136

After excluding the 21 co-positive isolates, only six *exoU*-positive (*exoS*-negative) isolates remained. The direction and magnitude of the association were preserved, but the small number limited statistical power and the comparison did not reach significance.
